# Controversies of antioxidant vitamins supplementation in exercise: ergogenic or ergolytic effects in humans?

**DOI:** 10.1186/1550-2783-11-4

**Published:** 2014-02-19

**Authors:** Cainara Lins Draeger, Andréia Naves, Natália Marques, Ana Beatriz Baptistella, Renata Alves Carnauba, Valéria Paschoal, Humberto Nicastro

**Affiliations:** 1 VP Research Institute, Rua Pedro Morganti, 103 – Vila Mariana, PO Box 04020-070, São Paulo, SP, Brazil

**Keywords:** Endurance, Oxidative stress, Antioxidant, Athlete, Diet, Physical activity, Vitamin A, Vitamin C, Vitamin E and β-caroten

## Abstract

The aim of this commentary was to discuss the last studies regarding the effect of antioxidant vitamins supplementation on oxidative stress in exercise in humans. The inclusion criteria encompassed published studies done in adult males and females between 2006 and 2013. The keywords used in the search engine were: endurance athlete, diet, oxidative stress, physical activity, diet, nutrition, antioxidant, antioxidant status, vitamin C, vitamin A, vitamin E, β-carotene and combinations. Twelve studies were identified and organized according to the methodology and results of supplementation: ergogenic, ergolytic, partial or no difference between groups. The results of these studies showed no effect on physiological parameters and activity of antioxidant enzymes (n = 07), better response of the placebo treatment (ergolytic effect; n = 02), partial results (n = 01) and ergogenic results of antioxidant supplementation (n = 02). It is concluded that supplementation with antioxidant vitamins has controversial effects to oxidative damage induced by endurance exercise. The discordances among the studies are presented and discussed.

## Background

Oxidative stress occurs when there is an imbalance in the human body homeostasis, i.e. the production of pro-oxidants becomes excessive and the cellular antioxidant mechanisms cannot neutralize these radicals. Excessive production of free radicals can be triggered by several endogenous and exogenous factors and, among these, exposure to radiation, excessive heat, inflammation, infection, trauma and exhaustive physical exercise can be considered strong exogenous triggers [1].

The regular practice of exercise induces several adaptations in cardiovascular, skeletal muscle and respiratory systems providing positive results for the prevention and treatment of metabolic diseases [2]. However, despite the undeniable health benefits, exercise may increase mitochondrial formation of reactive oxygen species which may cause cellular damage [3]. When produced in excess, free radicals may promote cellular oxidation, damage in the DNA structure, aging and a variety of diseases [4], impair skeletal muscle function and pain and, thereby affecting exercise performance [5]. In an attempt to minimize the effects of oxidative stress during physical activity, many athletes and sports professionals are performing supplementation with antioxidant vitamins.

However, recent studies raise the assumption that exercise alone could increase the oxidative capacity of skeletal muscle and potentiate the action of endogenous antioxidants, which is sufficient to counteract the negative effects of oxidative stress induced by the mechanical stimuli [3,6-8]. In view of this background, the aim of this commentary was to systematize the results of the last studies published regarding the effects of antioxidant vitamins intake on oxidative stress in exercise in humans.

## Results and discussion

We included 12 studies published in the last years that addressed the supplementation of antioxidant vitamins in trained volunteers (n = 05; Table 1) and in volunteers submitted to endurance exercise (n = 07; Table 2).

**Table 1:** Results of the studies with endurance trained volunteers supplemented with vitamins A, C, and E

**Study**	**Experimental design**	**Sample**	**Duration**	**Suplementation protocol**			**Result**	
				**Vitamin A**	**Vitamin C**	**Vitamin E**	**Ergogenic**	**Ergolytic**
Tauler et al. [6]	Randomized, double-blind	15 athletes	90 d*	30 mg	1000 mg	500 mg	↔	↔
				(β-caroten)				
Gauche et al. [9]	Randomized, double-blind	22 athletes	21 d (pre-exercise) + 2 dias (post-exercise)	6 mg	200 mg	32 mg	↑	N/R
				(β-caroten)				
Nielsen et al. [10]	Randomized, double-blind, cross-over	15 athletes	28 d	-	400 mg	180 mg	↔	↔
Patil et al. [11]	Randomized, double-blind	37 athletes	21 d	-	-	200 mg	↔	↔
Louis et al. [12]	Randomized, double-blind	16 athletes	21 d	17.1 mg	319.2 mg	48 mg	↑	N/R
				(β-caroten)				

* Vitamin C supplementation occurred only in the last 15 days of the study; ↑ Improved exercise performance; ↔ No results on exercise performance; N/R – not reported.

**Table 2:** Results of the studies with untrained volunteers submitted to endurance exercise and supplemented with vitamins C e E

**Study**	**Experimental design**	**Sample**	**Duration**	**Supplementation protocol**		**Result**	
				**Vitamin C**	**Vitamin E**	**Ergogenic**	**Ergolytic**
Bloomer et al. [13]	Randomized, double-blind	15 trained and e 15 untrained subjects	14 d (pre-exercise) + 2 d (post-exercise)	2000 mg	835 mg	↔	↔
Gomez-Cabrera et al. [7]	Randomized, double-blind	14 untrained subjects e 36 rats	8 weeks	1 g (humans) and 0.24 mg∙cm^-2^ (rodents)	-	N/R	**↓**
Ristow et al. [3]	Randomized, double-blind	20 trained and e 20 untrained subjects	4 weeks	1000 mg	440 mg	N/R	**↓**
Yfanti et al. [14]	Randomized, double-blind	21 untrained subjects	16 weeks	500 mg	400 IU	↔	↔
Yfanti et al. [5]	Randomized, double-blind	21 untrained subjects	16 weeks	500 mg	400 IU	↔	↔
Nalbant et al. [8]	Randomized	57 elderly	6 months		900 IU	↔	↔
Nakhostin et al. [15]	Randomized	16 untrained subjects	N/R	1000 mg	-	↑↓	N/R

↑ Improved exercise performance; **↓** Impaired exercise performance; ↑↓ Partial result; ↔ No results on exercise performance; IU – International Units; N/R – not reported.

In general, it was observed that there are controversial results about antioxidant supplementation during high-intensity exercise. According to two studies evaluated [3,7], the placebo group presented significant better physical performance, fatigue resistance and antioxidant protection when compared to the supplemented groups. In contrast, Gauche et al. [9] and Louis et al. [12] evaluated the effects of vitamin and mineral supplementation on muscle activity of athletes and observed that dietary supplementation provided a slight advantage over the placebo group in maximum voluntary muscle contraction after high-intensity exercise. This small advantage in the supplemented group compared to the placebo group was sufficient for the authors to consider the antioxidant supplementation as an ergogenic aid. Regarding the other studies, no differences were found between the groups.

### Sample characteristics

The subjects included in the studies presented different metabolic and body composition patterns. It is known that untrained subjects are more responsive to an exercise bout and, consequently, much more susceptible to suffer cellular damage from oxidative stress than trained individuals. For example, muscle damage caused by oxidative stress, in general, is more pronounced in untrained individuals [16].

Another point to be considered is the sample size of the studies. It was observed that the number of individuals that comprise the groups used in the studies listed in Table 1 is smaller than those in Table 2. This can be partially justified by the difficulty of recruiting athletes to be volunteers. Consequently, the statistical power and the effect size of such data can be compromised and should be carefully interpreted.

### Dietary control

Parallel to vitamin supplementation, it was observed that several studies did not perform dietary control of the subjects [3] or performed an inadequate control [9-12] to assess the possible interference of diet on the outcome. The dietary control is quite important since some vitamins and minerals may compete in terms of absorption in the gastrointestinal tract. Thus, the absence or inadequate dietary control can be considered a bias of the published studies.

Tauler et al. [6] and Yfanti et al. [5,14] performed dietary control through food records before and after the intervention. Gomez-Cabrera et al. [7] instructed the subjects to repeat the diet in the day before the exercise test in the pre- and post-supplementation periods. Only in the study of Bloomer et al. [13] dietary control was performed through food records. The variables analyzed were: total caloric value of the meals, amount of proteins, carbohydrates and lipids and of vitamins A, C and E. Even using a better dietary control than the other studies, the authors did not identify differences in the diet that could justify the results obtained.

### Controversies

The differences between the results in the studies described can also be mainly attributed to the different methodologies, conveyed vitamin dosage, study length, sample size, differences in gender, age, and subjects characteristics (athletes and non-athletes). These differences make it difficult to draw conclusion about the advantages and disadvantages of antioxidant vitamins supplementation.

So far, the results of the studies presented confirm that exercise is capable of increasing the oxidative capacity of skeletal muscle and potentiate the action of endogenous antioxidants [6]. Exercise increases the expression of reduced glutathione (GSH) and antioxidant enzymes (superoxide dismutase [SOD], and glutathione peroxidase [GSH-Px]), which appear to be sufficient to counteract the negative effects of exercise-induced oxidative stress [3,7,8]. In this context, the real need to use antioxidant vitamins supplements as ergogenic aids is questionable. The safest and effective alternative in attenuating exercise-induced oxidative stress could be a balanced diet based on foods with the recommended amounts of antioxidants in order to improve exercise performance.

## Conclusions

The results obtained in the considered studies with antioxidant vitamins supplementation are contradictory. Some studies show that supplementation does not improve exercise performance but can impair it. Others show that supplementation provides a slight advantage over the placebo. Thus, although many athletes use antioxidant supplementation to improve their physical performance, there is no consistent evidence suggesting that supplementation reduces oxidative stress and ensures better results in exercise.

## Abbreviations

DNA: Deoxyribonucleic acid; GSH: Reduced glutathione; GSH-Px: Glutathione peroxidase; SOD: Superoxide dismutase.

## Competing interests

The authors declare that they have no competing interests.

## Authors’ contributions

CLD participate in the manuscript design and wrote the first draft of the manuscript. AN, NM, ANB, RAC, and VP participated in the interpretation and preparation of the manuscript. HN participated in the manuscript design, interpretation and preparation of the manuscript. All the authors read and approved the final manuscript.
